# A single amino acid mutation alters substrate specificity towards l-glucose in a bacterial l-galactose dehydrogenase

**DOI:** 10.1016/j.bbrep.2026.102819

**Published:** 2026-10-02

**Authors:** Nana Obara, Chihiro Ito, Keito Kawashita, Mihoko Takenoya, Myoungjin Kim, Shinsaku Ito, Yasuyuki Sasaki, Akira Nakamura, Shunsuke Yajima

**Affiliations:** a Department of Bioscience, Tokyo University of Agriculture, Setagaya-ku, Tokyo, 156-8502, Japan; b Institute of Life and Environmental Sciences, University of Tsukuba, 1-1-1 Tennodai, Tsukuba, Ibaraki, 305-8572, Japan; c Microbiology Research Center for Sustainability (MiCS), University of Tsukuba, 1-1-1 Tennodai, Tsukuba, Ibaraki, 305-8572, Japan; d Tsukuba Institute for Advanced Research (TIAR), University of Tsukuba, 1-1-1 Tennodai, Tsukuba, Ibaraki, 305-8572, Japan

**Keywords:** l-Glucose dehydrogenase activity, Enantioselectivity, Substrate specificity, X-ray crystallography

## Abstract

Structure-based mutagenesis was performed to modify the substrate specificity of bacterial l-galactose dehydrogenase from *Luteolibacter* sp. strain LG18, which also exhibits intrinsic l-glucose dehydrogenase activity. Based on previously reported crystal structures of the enzyme in ternary complex with l-galactose or l-glucose and NADP^+^, an M212Q mutant was generated and found to exhibit a pronounced preference for l-glucose.

Kinetic analysis showed that the M212Q mutant displayed an approximately 20-fold higher *k*_cat_/*K*_M_ value for l-glucose than for l-galactose. To elucidate the structural basis of this altered specificity, the X-ray crystal structure of the M212Q mutant complexed with l-glucose and NADP^+^ was determined. Substitution of Met212 with Gln is assumed to disrupt the hydrophobic environment between the residue and the substrates, which may prevent proper binding of l-galactose and resulting in an approximately 350-fold increase in *K*_M_ compared with the wild type. In contrast, in the l-glucose–bound M212Q structure, a compensatory hydrogen bond is formed between the equatorial 4-OH group of l-glucose and Gln212, stabilizing substrate binding and reducing the *K*_M_ value to approximately one-quarter of that of the wild type. Furthermore, a BLAST search with Lu-LGDH as a query resulted that homologs containing the glutamine residue at the corresponding position as well as other conserved catalytic and substrate-binding residues were found.

With emerging studies on l-glucose biology, these results may contribute to enzymatic approaches for detecting the enzymes acting toward l-glucose, which is generally considered absent in nature.

## Highlights

• l-Galactose dehydrogenase from *Luteolibacter* sp. LG18 was investigated.
• The M212Q mutation showed strong substrate preference to l-glucose.
• Structural analysis revealed the role of the substituted glutamine residue.
• Homologous proteins containing glutamine at the M212 position were found in GenBank.

## Graphical abstract

**Figure:**
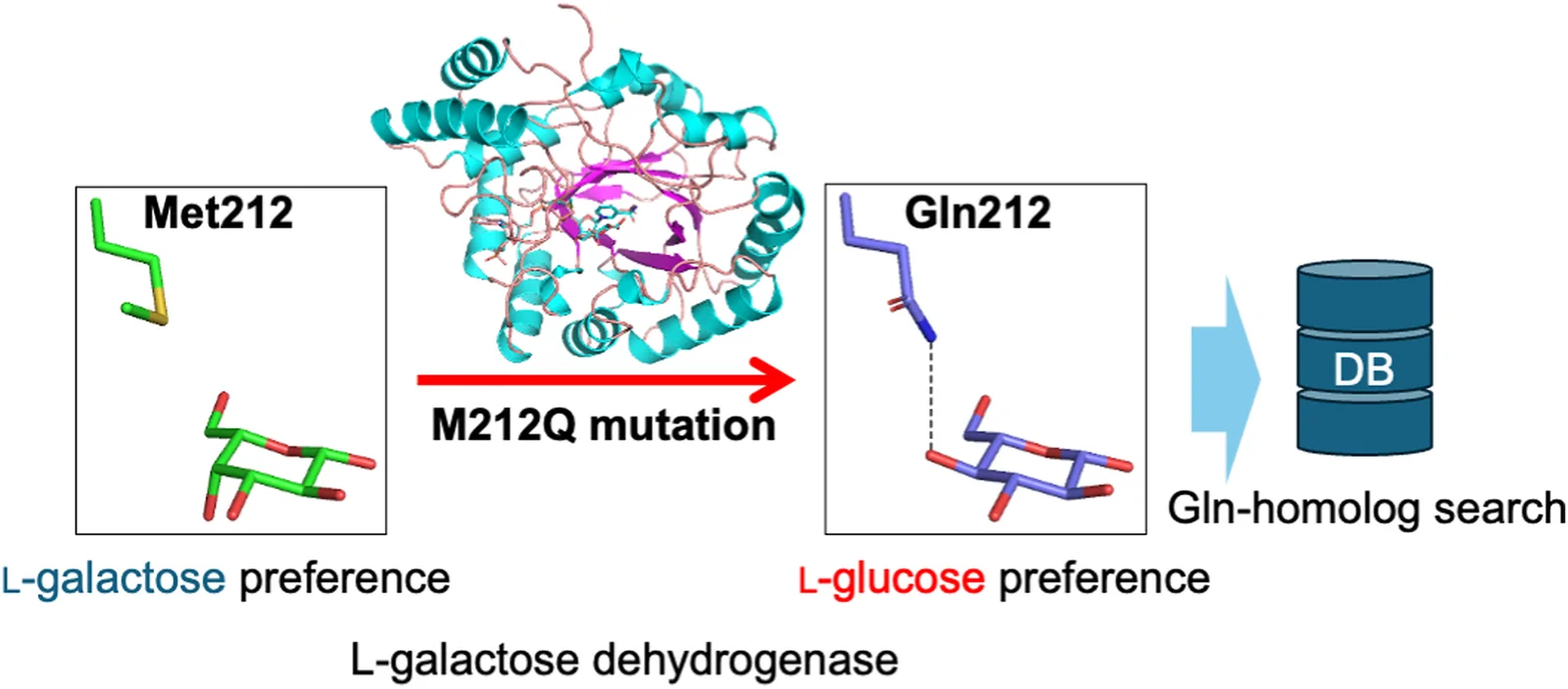

## 1 Introduction

In biological systems, the utilization of sugars and amino acids is generally restricted to homochiral compounds. In particular, glucose is considered to exist exclusively in the d-form in nature. l-Glucose has long been regarded as absent from natural environments, and early work reported that biological systems are unable to metabolize l-glucose [1].

However, this prevailing view has been challenged by subsequent studies, as several bacterial strains capable of growth on l-glucose as the sole carbon source have been isolated. It was shown that *Paracoccus laeviglucosivorans* grows on l-glucose as the sole carbon source, with the initial step of its l-glucose metabolism catalyzed by a *scyllo*-inositol dehydrogenase (Pl-*scyllo*-IDH) exhibiting l-glucose dehydrogenase activity [2]. More recently, *Luteolibacter* sp. strain LG18 was also demonstrated to utilize l-glucose, and its initial metabolic step was shown to be catalyzed by l-galactose dehydrogenase (Lu-LGDH) [3].

In addition to such metabolic studies, enzymatic activities related to l-glucose have also been reported. For example, a d-*threo*-aldose dehydrogenase from *Pseudomonas caryophylli* [4] and l-galactose dehydrogenase from *Phocaeicola vulgatus* (Bvu_0129) [5] were shown to catalyze reactions involving l-glucose. Furthermore, α-l-glucosidases were isolated from *Cecembia lonarensis* LW9 [6].

In order to elucidate the catalytic mechanisms for l-glucose, X-ray crystallographic studies have been reported in complex with substrates for some of enzymes above.

These enzymes—Pl-*scyllo*-IDH [7], Lu-LGDH [8], and the α-l-glucosidase [6]—belong to distinct enzyme superfamilies, and the substrate recognition modes vary accordingly.

l-Galactose dehydrogenases (LGDH) have been well studied in plants (EC 1.1.1.316) because they play a key role in ascorbic acid biosynthesis [9,10]. Plant LGDHs catalyze the NAD^+^-dependent oxidation of the C1 hydroxyl group of l-galactose to form l-galactono-1,4-lactone, a key intermediate in ascorbic acid biosynthesis. Crystal structures of apo forms and cofactor-bound complexes have been reported for enzymes from rice (*Oryza sativa*) [11], spinach (*Spinacia oleracea*) [12], and camu-camu (*Myrciaria dubia*) [13]. In contrast, bacterial LGDHs (EC 1.1.1.x) remain less characterized, and no structural information was available before our previous report [8]. Hobbs et al. reported that the LGDH from *P. vulgatus* catalyzes the NADP ^+^ -dependent oxidation of l-galactose to l-galactono-1,5-lactone, which is subsequently metabolized by lactonase and galactonate dehydrogenase [5]. Yachida et al. demonstrated that Lu-LGDH functions as the first enzyme in a catabolic pathway leading to the formation of pyruvate and d-glyceraldehyde 3-phosphate from l-galactose [3].

Recently, we reported the crystal structures of Lu-LGDH in ternary complexes with l-galactose or l-glucose and NADP^+^. These structural analyses revealed that the 4-OH groups, the only structural difference between the two pyranoses, are not involved in the substrate binding in the active site [8]. Despite this similarity, the *K*_M_ values for both sugars differ markedly, being approximately 0.24 mM and 14 mM for l-galactose and l-glucose, respectively. Based on the crystal structures of the ternary complexes, this difference is expected to arise from interactions between the 4-OH group and hydrophobic residues, Met212 and Trp223. In the native substrate, l-galactose, the hydrophilic 4-OH group adopts an axial orientation that protrudes away from the hydrophobic residues Met212 and Trp223 and lies within hydrogen-bonding distance of at least two ordered water molecules. In contrast, the equatorial orientation of the O4 hydroxyl group in l-glucose places it closer to Met212 and Trp223, which may disrupt these water-mediated interactions without providing compensatory protein-mediated hydrogen bonds.

Based on this structural insight, we prepared two mutants, M212Q and W223F to modulate the local environment surrounding the 4-OH group and evaluate their effects on substrate specificity.

Here, we report that a single amino-acid substitution confers higher specificity toward l-glucose than the wild-type enzyme, and the X-ray crystal structure of the mutant in complex with l-glucose and NADP^+^ provides structural insight into the molecular basis of the altered substrate specificity. Furthermore, a BLAST search with Lu-LGDH as a query resulted that homologs containing the mutated residue was performed.

The findings of this study add further support to previous reports suggesting the potential involvement of l-glucose in biological systems and this mutant may contribute to the development of enzymatic tools for exploring the presence of l-glucose in nature.

## 2 Materials and methods

### 2.1 Protein expression and purification

The pET28a(+) construct carrying the Lu-LGDH gene described in our previous study was used for recombinant protein production [8]. *Escherichia coli* Rosetta 2(DE3) transformed with this plasmid was cultivated in LB medium containing 34 μg/mL chloramphenicol and 50 μg/mL kanamycin at 37 °C. Protein expression was induced with 200 μM isopropyl β-d-1-thiogalactopyranoside, and cultivation was continued for an additional 3 h at 30 °C.

Cells were harvested and resuspended in buffer A consisting of 20 mM Tris–HCl (pH 7.5), 300 mM NaCl, and 10% glycerol. Following cell disruption by sonication, insoluble material was removed by centrifugation at 69,673 × g for 35 min. The resulting supernatant was applied to a Ni-NTA affinity column (Qiagen), and bound protein was recovered using buffer A supplemented with 500 mM imidazole. Eluted fractions were dialyzed against buffer A, and the enzyme preparation was concentrated with an Amicon Ultra-4 Ultracel-10K device (Millipore).

### 2.2 Introduction of single amino acid mutation

Single amino acid substitutions of M212Q and W223F were carried out by an inverse PCR method using Tks Gflex DNA polymerase (Takara Bio Inc.) with the corresponding primer sets (M212Q: forward 5′-TCGCCGACGGGCCAGGGGCTGCTGAC, reverse 5′- TCAGCAGCCCCTGGCCCGTCGGCGAG: W223F: forward 5′- GCGCGCCTTCGTTTCATCCGTCCTC, reverse 5′- AGGACGGATGAAACGAAGGCGCGCC: mutated codons were underlined.) and the Lu-LGDH gene in pET28a(+) as a template. The sequence of each mutated gene was confirmed using Sanger sequencing (Macrogen Japan Corp.).

### 2.3 Kinetic analysis

The catalytic activities of wild-type Lu-LGDH and its variants were determined by monitoring NADPH formation at 340 nm at 25 °C. NADP^+^ was obtained from Roche, whereas l-galactose and l-glucose were purchased from Tokyo Chemical Industry Co., Ltd. (TCI).

Assays were carried out in 20 mM Tris–HCl buffer (pH 7.5) containing 1.0 mM NADP^+^, substrate, and enzyme. Depending on the substrate and enzyme variant examined, substrate concentrations were adjusted between 0.05 and 50 mM. Reactions were initiated by addition of the enzyme to a continuously stirred cuvette, and reaction rates were determined from the initial linear phase within the first 60 s.

NADPH production was quantified using an extinction coefficient of 6220 M^−1^ cm^−1^. Kinetic parameters were estimated by fitting the data to the Michaelis–Menten equation using the analysis software provided with a V-750 spectrophotometer (JASCO, Tokyo, Japan).

### 2.4 Crystallization, data collection, and structure determination

Crystallization trials were performed by the hanging-drop vapor-diffusion method, in which 1 μL of protein solution was mixed with an equal volume of reservoir solution. Initial crystallization conditions were screened using commercially available kits, and promising conditions were subsequently optimized.

For preparation of the ternary complex of the Lu-LGDH M212Q mutant with l-glucose and NADP^+^, the protein solution was supplemented with 50 mM l-glucose and 10 mM NADP^+^ before setting up crystallization drops. Crystals suitable for diffraction experiments were obtained from a reservoir solution composed of 30% (w/v) PEG 8000 and 200 mM ammonium sulfate.

X-ray diffraction data were collected at 95 K under a nitrogen cryostream on beamline BL-1A at the Photon Factory (KEK). Image processing and scaling were performed using the onsite automated pipeline incorporating XDS [14], followed by Pointless [15] and Aimless [15] from the CCP4 package [16].

The structure was solved by molecular replacement with Molrep [17] using the previously determined the wild type of Lu-LGDH complexed with l-glucose and NADP^+^ (PDB ID: 9W7A) as the search model. Refinement was initiated in Phenix [18], including a simulated-annealing step, and was further improved through alternating cycles of refinement in Refmac5 [19] and manual model building with Coot [20].

Crystallographic statistics are summarized in Supplementary Table 1. Coordinates and structure factors for the M212Q mutant complexed with l-glucose and NADP^+^ have been deposited in the Protein Data Bank under accession code 26WE. Molecular graphics were prepared with the PyMOL molecular visualization package (Schrödinger LLC).

## 3 Results and discussion

### 3.1 Kinetic analysis of mutant enzymes

Based on the previously reported crystal structure [8], the lower affinity of l-glucose was hypothesized to arise from the orientation of its 4-OH group toward Met212 and Trp223, which may disrupt favorable water-mediated interactions without providing compensatory protein-mediated hydrogen bonds in the active site of Lu-LGDH. Therefore, we attempted to enhance the affinity of Lu-LGDH for l-glucose by introducing amino acid substitutions. Glutamine was selected to introduce a neutral hydrophilic side chain of similar length to methionine, whereas phenylalanine was selected to retain hydrophobicity while reducing the size of the aromatic side chain relative to tryptophan. We prepared two mutant enzymes, M212Q and W223F, and compared their enzymatic activities toward l-galactose and l-glucose with those of the wild-type enzyme in order to improve affinity for l-glucose. As summarized in Table 1 and Supplementary Fig. 1, the M212Q and W223F mutants exhibited distinct kinetic behaviors.

**Table 1:** Kinetic parameters of the wild type and mutants of Lu-LGDH.

Substrate	Enzyme	*K*_M_ (mM)	*k*_cat_ (s^−1^)	*k*_cat_/*K*_M_ (s^−1^mM^−1^)
L-galactose	WT	2.82 × 10^−1^ ± (5.1 × 10^−2^)	14.7 ± 2.1	52.5 ± 3.44
	M212Q	1.01 × 10^2^ ± 31	13.1 ± 3.4	1.30 × 10^−1^ ± (5.5 × 10^−3^)
	W223F	2.89 ± (4.7 × 10^−1^)	94.7 ± 13	32.9 ± 3.4
L-glucose	WT	20.8 ± 2.9	65.0 ± 7.6	3.15 ± (2.7 × 10^−1^)
	M212Q	5.53 ± (9.2 × 10^−1^)	15.4 ± 2.6	2.79 ± (3.3 × 10^−2^)
	W223F	1.56 × 10^2^ ± 42	3.35 × 10^2^ ± 79	2.16 ± (7.3 × 10^−2^)

The M212Q mutant exhibited, toward l-galactose as a substrate, the *k*_cat_ value comparable to the wild type and the increased *K*_M_ value by approximately 350-fold. In contrast, for l-glucose, the *k*_cat_ value of M212Q was reduced to approximately 4-fold lower than that of the wild type; however, the *K*_M_ value was also decreased by approximately 4-fold. Therefore, this mutation gave a reduced catalytic efficiency about 400-fold toward l-galactose, while remained almost unchanged toward l-glucose.

For the W223F mutant, when l-galactose was used as the substrate, the *K*_M_ value increased by approximately 10-fold relative to the wild-type enzyme, while the *k*_cat_ value increased by approximately 7-fold. When l-glucose was used as the substrate, the *K*_M_ value was increased by approximately 7.5-fold and the *k*_cat_ value was also increased by approximately 6-fold compared with the wild type. Thus, the W223F mutant exhibited similar kinetic efficiency toward both sugars. Although slight differences were observed in the *K*_M_ and *k*_cat_ values compared with those reported previously [8], we consider that the overall trends in substrate preference and catalytic behavior remain consistent between the two studies.

Based on the results described above, Trp223 appears to play a more general role in maintaining proper substrate positioning within the active site. In contrast, Met212 is located above the planar face of the sugar ring and contributes to hydrophobic interactions with the substrate. We hypothesized that replacement of Met212 with a glutamine residue would enable the formation of a hydrogen-bonding interaction between the 4-OH group of l-glucose and the introduced side chain.

The reduced *K*_M_ value for l-glucose observed in the M212Q mutant is consistent with the proposed interaction; however, because *K*_M_ is not necessarily equivalent to substrate-binding affinity, the present kinetic data alone do not prove its formation. To further clarify the structural basis of this altered substrate recognition, we next sought to determine the crystal structure of the ternary complex of the M212Q mutant with l-glucose and NADP^+^.

### 3.2 Structural analysis of the ternary complex of the M212Q mutant with l-glucose and NADP^+^

A crystal of the ternary complex of the Lu-LGDH M212Q mutant with l-glucose and NADP^+^ was obtained in the space group *P*2_1_2_1_2_1_ and contained two monomers per asymmetric unit. The crystallization condition comprised 30% (w/v) PEG 8000 and 200 mM ammonium sulfate, which was identical to the one used for the wild-type enzyme [8].

Initial structure was obtained by molecular replacement using the previously determined wild-type Lu-LGDH/l-glucose/NADP^+^ ternary complex (PDB ID: 9W7A) as a search model. Refinement of the structure converged at a resolution of 1.76 Å, with final *R* and *R*_free_ values of 0.185 and 0.204, respectively (Supplementary Table 1). As shown in Fig. 1, the polder maps clearly reveal electron density corresponding to l-glucose and NADP^+^. However, no clear electron density was observed for the nicotinamide ring of NADP^+^, as the case in the wild-type structure.

**Fig. 1:**
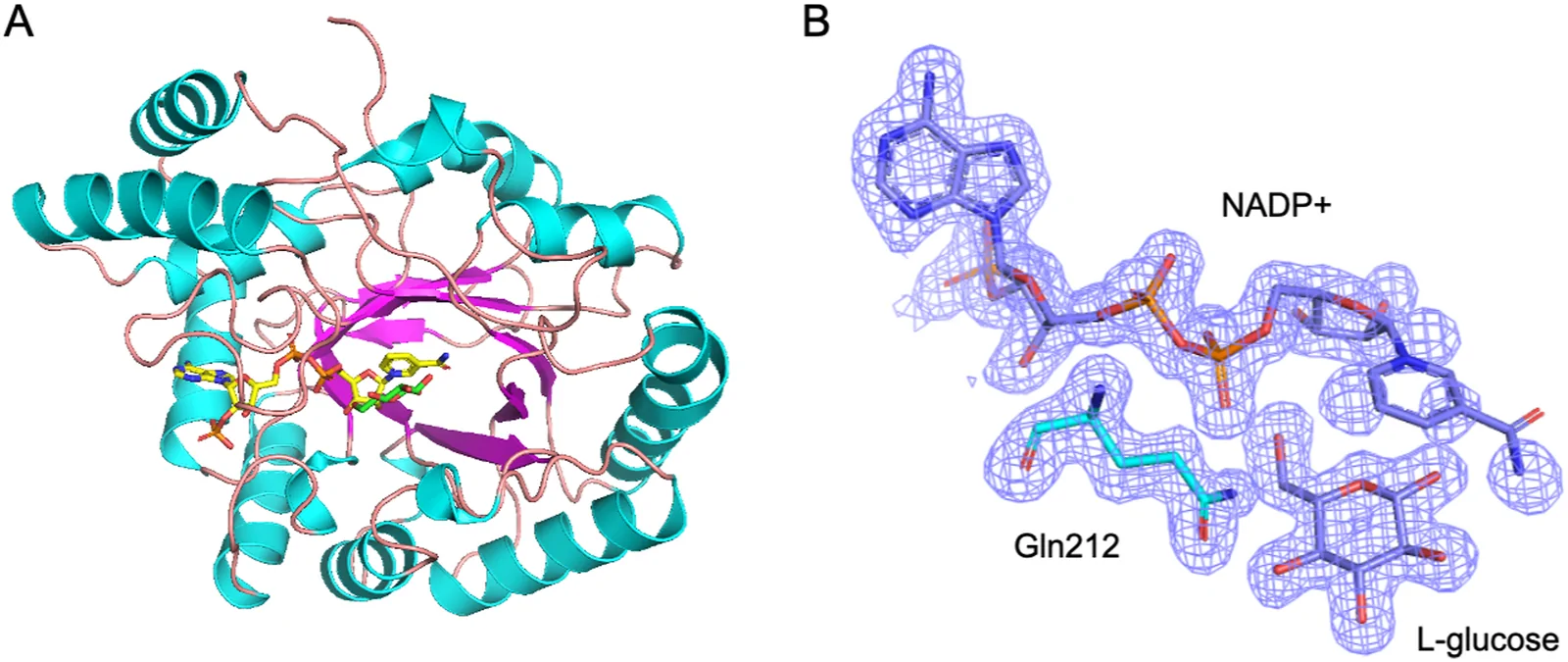
Overall structure of the M212Q mutant of Lu-LGDH in complex with l-glucose and NADP^+^. (A) Overall structure is shown as a cartoon representation colored according to secondary structure. l-glucose and NADP^+^ are presented as stick models in green and yellow, respectively. (B) A polder map is contoured at 3σ is shown for Gln212, l-glucose and NADP^+^.

The structures of the wild-type enzyme and the M212Q mutant were superposed using the MatchMaker function in UCSF Chimera [21]. The overall protein structures were well aligned with a root-mean-square deviation (RMSD) of 0.120 Å over 326 aligned residues, and the cofactor, the substrate, and amino acid residues involved in substrate binding also showed good overlap. In addition, the side chains of Met212 in the wild-type enzyme and Gln212 in the mutant were well aligned (Fig. 2A).

**Fig. 2:**
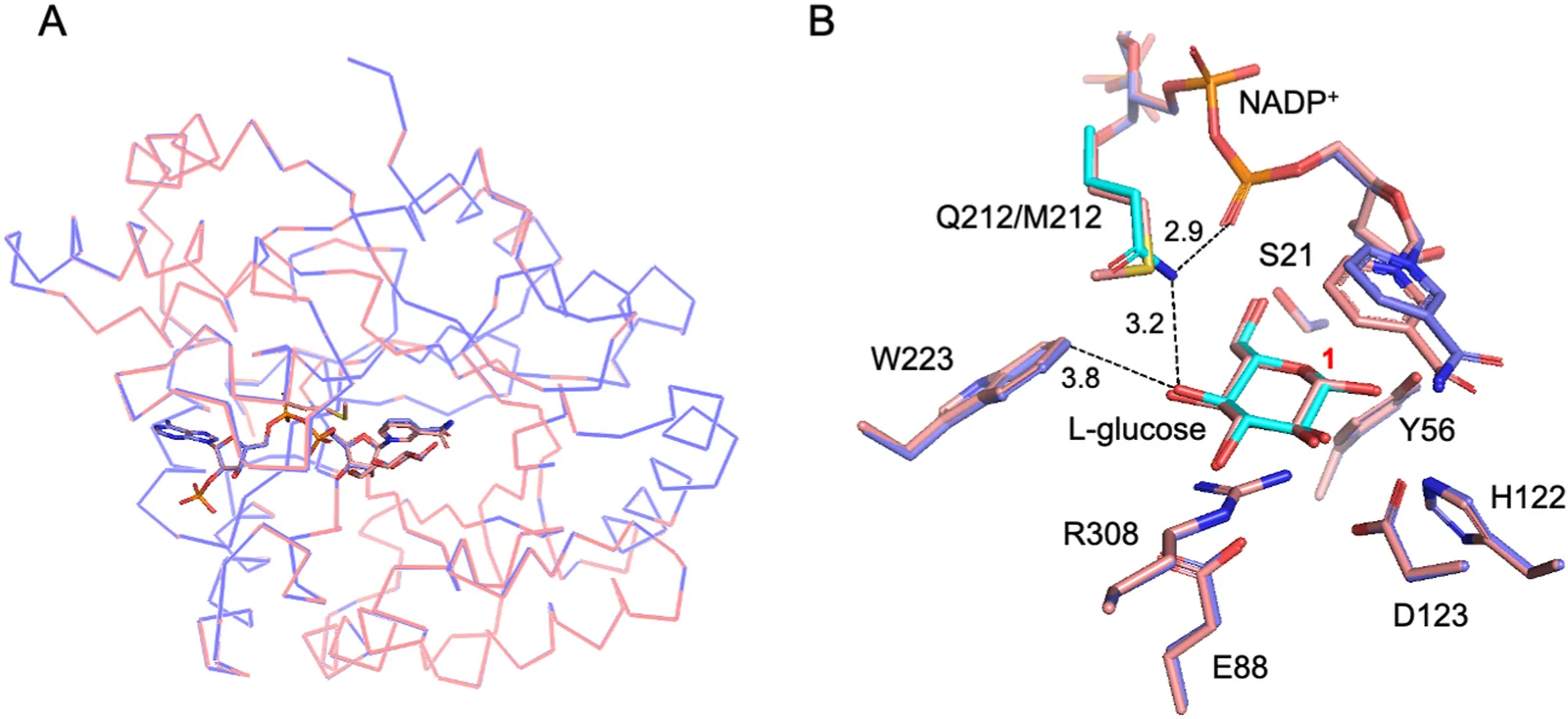
Superposition of the wild type and M212Q mutant of Lu-LGDH. The structures of the wild type and M212Q mutant of Lu-LGDH were superposed using UCSF Chimera. The wild type and mutant are colored pink and blue, respectively. (B) Close-up view of the active site architecture in the superposed structures. Gln212 and l-glucose shown in cyan correspond to those in the mutant enzyme. The numbers along with dotted lines show the distances in Å. The red number 1 indicates the C1 position of l-glucose.

The distance between the terminal amide group of the Gln212 side chain and the 4-OH group of l-glucose was measured to be 3.2 Å, a geometry that is compatible with the formation of a hydrogen bond (Fig. 2B). In the wild type, Met212 is located above the planar face of the sugar ring and contributes to hydrophobic interactions with the substrates. This interaction is important for positioning both l-galactose and l-glucose within the active site. Loss of this hydrophobic interaction could lead to unfavorable substrate binding in the active site. On the other hand, the introduced glutamine residue appears to promote proper binding of l-glucose through the formation of a hydrogen bond with the equatorial 4-OH group, which represents the only structural difference from l-galactose.

The two independent molecules (chains A and B) in the asymmetric unit exhibited essentially identical active-site structures. In both chains, in addition to the hydrogen-bonding interaction between Gln212 and the 4-OH group of l-glucose, the side-chain amide group of Gln212 forms a hydrogen-bonding interaction with the β-phosphate group of NADP^+^ (Fig. 2B, Supplementary Fig. 2). Furthermore, a conserved water molecule was observed between Gln212 and the 4-OH group of l-glucose (Supplementary Fig. 2).

Although the side-chain amide group of Gln212 lies within hydrogen-bonding distance of the β-phosphate group of NADP^+^ and may therefore interact with the cofactor (Fig. 2B), the observed disorder of the electron density of the nicotinamide ring described above is unlikely to be a consequence of the M212Q substitution alone. The wild-type and M212Q ternary complexes revealed that not only the overall backbone structures but also the positions of l-glucose and NADP^+^ were essentially identical (Fig. 2), and the electron density corresponding to the nicotinamide moiety was weak in both the wild type [8] and the M212Q mutant complexes.

On the other hand, Trp223 plays a critical role in maintaining the correct positioning of the substrate in the active site by providing a hydrophobic environment. In support of this, a protein BLAST (blastp) search using the amino acid sequence of Lu-LGDH as a query showed that 99.3% of the top 1000 hits retain tryptophan at the corresponding position. Nevertheless, only the M212Q and W223F mutants were examined in the present study. Other amino acid substitutions may exert different effects on substrate recognition and catalysis, and further studies will be required to evaluate these possibilities.

### 3.3 Homolog search of the M212Q mutant

Since the M212Q mutation improved the affinity for l-glucose, we examined whether any homologous proteins containing a glutamine residue at the position corresponding to M212 exist in database. A blastp search was performed against the clustered non-redundant (nr) database using the amino acid sequence of Lu-LGDH as a query, and the top 1000 hits were retrieved. Among these, sequences containing glutamine at the corresponding position were selected and used as queries for subsequent blastp searches. This procedure was repeated for three rounds, resulting in the identification of 20 sequences, including two partial sequences, harboring glutamine at the corresponding position.

Most of these sequences were derived from metagenome-assembled genomes originating from soil environments [[22], [23], [24]], with three sequences annotated in RefSeq (WP_146601372 from *Novipirellula aureliae*, WP_058189412 from *Occallatibacter gabretensis*, and WP_424166923 from *Occallatibacter* sp.). Notably, the majority of these homologs were derived from the phylum *Acidobacteriota* (16 sequences) including *Occallatibacter*, with the remaining three belonging to the phylum *Planctomycetota* including *Novipirellula* (Supplementary Fig. 3).

Although the metagenome-assemble sequence may not be fully reliable, all of the identified proteins including the sequences from RefSeq conserved the catalytic tetrad characteristic of the aldo-keto reductase (AKR) superfamily, corresponding to Asp51, Tyr56, Lys82, and His122 in Lu-LGDH. In addition, these proteins also conserved residues implicated in substrate binding, including Ser21, Asp123, and Trp223. The preservation of these key residues suggests that these homologs are likely to retain catalytic competence and similar active-site architectures (Supplementary Fig. 4).

Furthermore, structural models predicted by ColabFold [25] for representative homologs showed close agreement with the Lu-LGDH structure upon superposition, not only in the conserved catalytic residues but also in the overall backbone architecture (Fig. 3). The superposition yielded a root mean square deviation of approximately 0.9 Å, calculated over 270 - 300 aligned residues, while the total sequence lengths ranged from 309 to 317 residues depending on the enzymes.

**Fig. 3:**
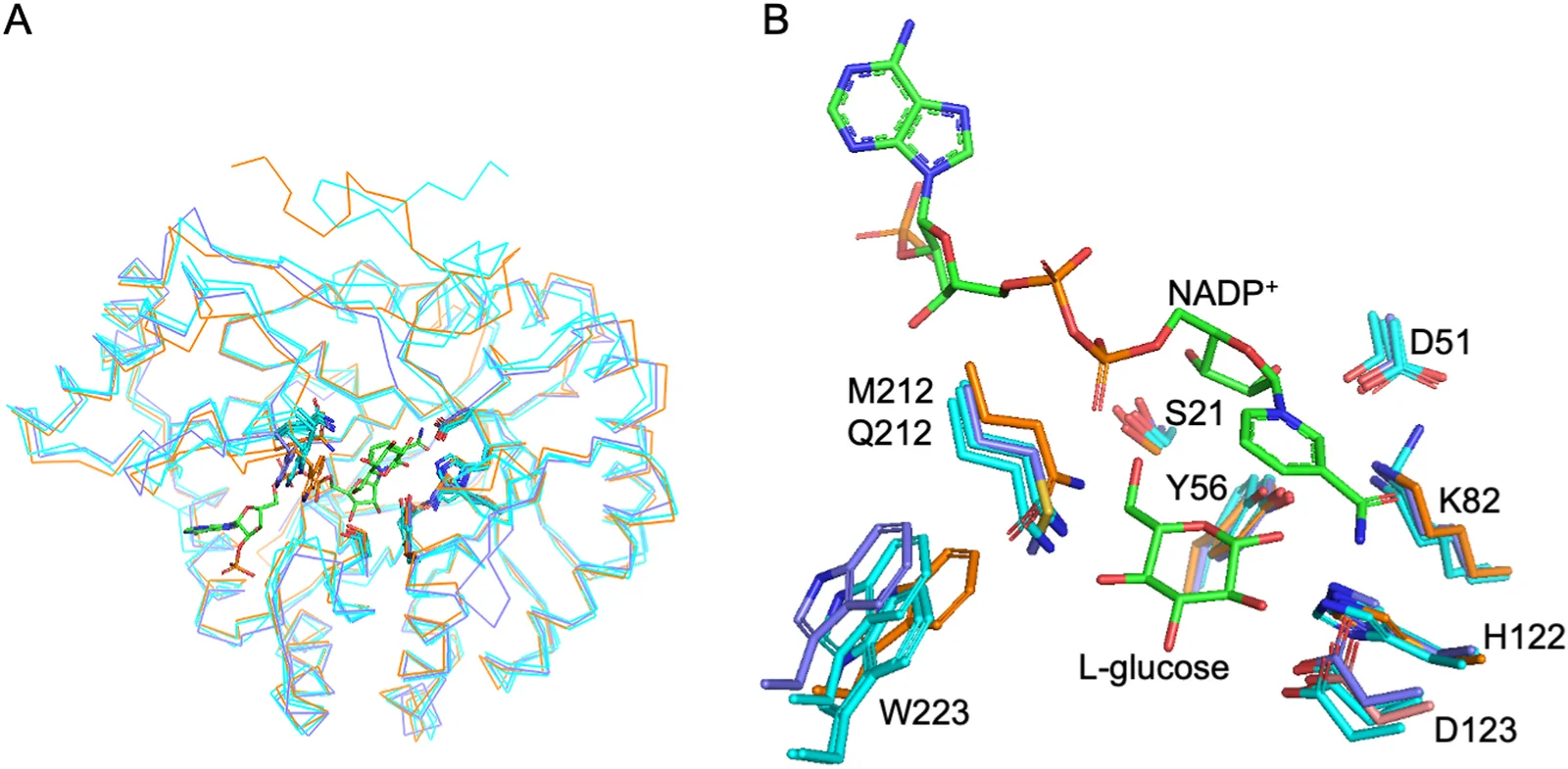
Superposition of the M212Q mutant of Lu-LGDH and ColabFold-predicted structures of the homologs. (A) The crystal structures of the M212Q mutant of Lu-LGDH, ColabFold-predicted structures of l-galactose dehydrogenase from *Phocaeicola vulgatus* (Pv-LGDH), and homologs containing glutamine at the corresponding position (GenBank IDs: HTF69926, WP_058189412, and WP_146601372) were superposed using UCSF Chimera. The phylogenetic relationships and sequence alignment among these proteins are shown in Supplementary Figs. 3 and 4, respectively. Lu-LGDH, Pv-LGDH and other predicted structures are colored orange, blue and cyan, respectively. l-glucose and NADP^+^ in Lu-LGDH are shown in a stick model colored green, and other important conserved residues are shown in a stick model colored the same as backbones. (B) The close-up view of the active site architecture in the superposed structures. The same colors are used as panel A. Pv-LGDH have methionine at the Met212 position in Lu-LGDH, and reported to exhibit l-glucose dehydrogenase activity.

These observations suggest that enzymes harboring glutamine at this position may constitute a distinct functional subgroup within LGDH-like proteins. Although experimental validation is required, it is conceivable that microorganisms possessing such enzymes could have an enhanced capacity to utilize l-glucose or structurally related sugars in natural environments.

Recently, α-l-glucosidases from *Cecembia lonarensis* LW9 have been reported [6], and a naturally occurring l-glucoside from *Homalium stenophyllum* has been identified by revising the structural assignment of a previously reported d-glucoside [26]. Furthermore, enrichment cultures with several atypical sugars, including l-glucose, using soil and activated sludge as inocula, revealed the phylogenetic diversity of bacteria capable of utilizing of l-glucose [27]. Therefore, although l-glucose has long been considered absent from natural systems, the results obtained in this study may also contribute to the exploration of l-glucose–related metabolism in nature.

## 4 Structure deposition

The atomic coordinates and the structure factors for M212Q mutant of l-galactose dehydrogenase from *Luteolibacter* sp. LG18 has been deposited in the Protein Data Bank with PDB ID 26WE.

## CRediT authorship contribution statement

**Nana Obara:** Investigation, Formal analysis, Data curation. **Chihiro Ito:** Investigation, Formal analysis, Data curation. **Keito Kawashita:** Investigation, Formal analysis, Data curation. **Mihoko Takenoya:** Investigation, Data curation. **Myoungjin Kim:** Investigation, Formal analysis, Data curation. **Shinsaku Ito:** Writing – review & editing, Supervision, Methodology. **Yasuyuki Sasaki:** Writing – review & editing, Supervision, Methodology. **Akira Nakamura:** Writing – review & editing, Resources, Methodology, Conceptualization. **Shunsuke Yajima:** Writing – review & editing, Writing – original draft, Visualization, Validation, Supervision, Project administration, Methodology, Formal analysis, Data curation, Conceptualization.

## Data availability statement

The data underlying this article will be shared at reasonable request to the corresponding author.

## Declaration of generative AI and AI-assisted technologies in the manuscript preparation process

The authors used Microsoft Copilot with Enterprise Data Protection in order to improve the English language and readability of the manuscript. After using this tool, the authors reviewed and edited the content as needed and take full responsibility for the content of the published article.

## Funding sources

This research did not receive any specific grant from funding agencies in the public, commercial, or not-for-profit sectors.

## Declaration of competing interest

The authors declare that they have no known competing financial interests or personal relationships that could have appeared to influence the work reported in this paper.

## Data Availability

Data will be made available on request.
