# Supplementary material for: A single amino acid mutation alters substrate specificity towards l-glucose in a bacterial l-galactose dehydrogenase
Source: Biochem Biophys Rep. 2026 Oct 2;48:102819. doi: 10.1016/j.bbrep.2026.102819 (PMC13661200; doi:10.1016/j.bbrep.2026.102819)
Supplement: Supplementary file 1 [file mmc1.docx]

Supplementary Information

A single amino acid mutation alters substrate specificity towards l-glucose in a bacterial l-galactose dehydrogenase

Nana Obara ^a,1^, Chihiro Ito ^a,1^, Keito Kawashita ^a^, Mihoko Takenoya ^a,2^, Myoungjin Kim ^a^, Shinsaku Ito ^a^, Yasuyuki Sasaki ^a^, Akira Nakamura ^b,c,d^, Shunsuke Yajima ^a,^*

^a^Department of Bioscience, Tokyo University of Agriculture, Setagaya-ku, Tokyo 156-8502, Japan

^b^Institute of Life and Environmental Sciences, University of Tsukuba, 1-1-1 Tennodai, Tsukuba, Ibaraki 305-8572, Japan.

^c^Microbiology Research Center for Sustainability (MiCS), University of Tsukuba, 1-1-1 Tennodai, Tsukuba, Ibaraki 305-8572, Japan.

^d^Tsukuba Institute for Advanced Research (TIAR), University of Tsukuba, 1-1-1 Tennodai, Tsukuba, Ibaraki 305-8572, Japan.

^1^These authors contributed to the work equally.

^2^Present address: Graduate School of Arts and Sciences, The University of Tokyo, Komaba 3-8-1, Meguro-ku, Tokyo 153-8902

*Corresponding author: yshun@nodai.ac.jp (S. Yajima)

Supplementary Table 1

Supplementary Figures 1 - 4

**Supplementary Table 1**. Data collection and Refinement statistics

|  | M212Q mutant of Lu-LGDH/l-glucose/NADP^+^ |
| --- | --- |
| **Data collection** |  |
| Beamline | PF BL1A |
| Wavelength (Å) | 1.0 |
| Detector | Eiger X 4M |
| Space group | *P*2_1_2_1_2_1_ |
| Unit cell parameters (Å) | *a* = 80.1 *b* = 88.8, *c* = 95.2 |
| No. of mol in ASU | 2 |
| Resolution range (Å) | 47.6 – 1.76 (1.79 – 1.76) |
| Completeness (%) | 100.0 (100.0) |
| *I*/σ(*I*) | 13.0 (2.2) |
| No. of unique reflections | 67918 (3807) |
| Multiplicity | 13.8 (13.3) |
| *R*_merge_ (%) | 0.162 (1.259) |
| CC(1/2) | 0.998 (0.764) |
|  |  |
| **Refinement** |  |
| Resolution range (Å) | 47.6 – 1.76 (1.81 – 1.76) |
| No. of reflections | 64487 (4917) |
| Completeness (%) | 100.0 (100.0) |
| *R*_work_/*R*_free_ | 0.185/0.204 (0.250/0.306) |
| No. of non-H atoms |  |
| Protein | 4977 |
| Ligand | 24 (l-glucose), 96 (NADP^+^), 5 (SO_4_) |
| Solvent | 437 |
| R.m.s. deviation |  |
| Bonds (Å) | 0.010 |
| Angle (°) | 1.553 |
| Average B factor (Å^2^) |  |
| Protein | 18.4 |
| Ligand | 16.8 (l-glucose), 19.1 (NADP^+^), 22.4 (SO_4_) |
| Solvent | 24.1 |
| Ramachandran plot (%) |  |
| Favored | 97.7 |
| Allowed | 2.3 |
| Disallowed | 0.0 |

Ramachandran plot was calculated by Rampage [1] in the CCP4 suite.

Reference

[1] S.C. Lovell, I.W. Davis, W.B. Arendall III, P.I.W. de Bakker, J.M. Word, *et al*., Structure validation by Calpha geometry: phi, psi and Cbeta deviation, Proteins: Struct. Funct. Genet., 50 (2003) 437-450, https://doi.org/https://doi.org/10.1002/prot.10286.

|  | **WT** (1 nM) | **M212Q** (5 nM) |
| --- | --- | --- |
| **l-galactose** |   *K*_M_ = 0.282  *k*_cat_ = 14.7 |   *K*_M_ = 101  *k*_cat_ = 13.1 |
| **l-glucose** |   *K*_M_ = 20.8  *k*_cat_ = 65.0 |   *K*_M_ = 5.53  *k*_cat_ = 15.4 |

**Supplementary Figure 1**

**s-v plots for the forward reactions of the wild type (WT) and the M212Q mutant using l-galactose and l-glucose as substrates.**

The concentrations of the enzymes used in the corresponding assays are indicated in parentheses next of the enzyme names. The solid lines represent nonlinear regression fits calculated to the Michaelis-Menten equation generated using KaleidaGraph software (Synergy Software). Error bars represent the standard deviations of three independent measurements. The values of *K*_M_ and *k*_cat_ are shown in the plots for reference; however, their standard deviations and units are omitted to avoid visual clutter. *K*_M_ and *k*_cat_ are expressed in mM and s⁻¹, respectively.

Detailed experimental procedures are described in the Materials and Methods section, and the kinetic parameters are summarized in Table 1 in the main text.

**Supplementary Figure 2**

**Superposition of the active sites of chains A and B in the M212Q-l-glucose-NADP⁺ ternary complex.**

The two molecules in the asymmetric unit were superposed using UCSF Chimera. Chains A and B are shown in green and blue, respectively. l-Glucose, NADP⁺, Gln212, and Trp223 are shown as stick models. The conserved water molecule is shown as a small sphere. Dashed lines indicate putative hydrogen-bonding interactions. Distances (Å) are shown next to the dashed lines. Where two values are given, the value outside parentheses corresponds to chain A, whereas the value in parentheses corresponds to chain B. The superposition demonstrates that the active-site architectures are essentially identical in the two chains and that the interactions involving Gln212, l-glucose, NADP⁺, and the conserved water molecule are preserved in both active sites.

**Supplementary Figure 3**

**Phylogenetic tree of Lu-LGDH homologs containing a glutamine residue at the position corresponding to Met212 in Lu-LGDH.**

Amino acid sequences of Lu-LGDH homologs identified by protein BLAST and containing glutamine at this position were aligned using ClustalW, followed by phylogenetic analysis with PhyML and 100 round bootstrap replicates on the Genome.jp server (<https://clustalw.genome.jp>). Proteins are labeled by their GenBank accession numbers. ABR37946 from *Phocaeicola vulgatus* contains methionine at this position and exhibits l-glucose dehydrogenase activity. The accession numbers with strain names highlighted in red were used for sequence alignment in Supplementary Figure 4 and for structural superposition of predicted models, as shown in Fig. 3 of the main text.

**Supplementary Figure 4**

**Sequence alignment of Lu-LGDH, Pv-LGDH and other homologs containing the glutamine residue.**

Five sequences (*Luteolibacter*; WP_338285331, *P. vulgatus*; ABR37946, *Edaphobacter*; HTF69926, *O. gabretensis*; WP_058189412, *N. aureliae*; WP_146601372) selected in Supplementary Figure 3 were aligned using ClustalW. Red inverted triangles are the conserved catalytic tetrad of the aldo/keto reductase superfamily. Green inverted triangles indicate other conserved residues involved in substrate recognition in Lu-LGDH. A blue circle indicate the methionine/glutamine residues highlited in this study.
